# Comparison of different inspiratory triggering settings in automated ventilators during cardiopulmonary resuscitation in a porcine model

**DOI:** 10.1371/journal.pone.0171869

**Published:** 2017-02-10

**Authors:** Dingyu Tan, Jun Xu, Shihuan Shao, Yangyang Fu, Feng Sun, Yazhi Zhang, Yingying Hu, Joseph Walline, Huadong Zhu, Xuezhong Yu

**Affiliations:** 1 Department of Emergency, Peking Union Medical College Hospital, Chinese Academy of Medical sciences, Beijing, China; 2 Division of Emergency Medicine, Department of Surgery, Saint Louis University Hospital, Saint Louis, Missouri; Azienda Ospedaliero Universitaria Careggi, ITALY

## Abstract

**Background:**

Mechanical ventilation via automated in-hospital ventilators is quite common during cardiopulmonary resuscitation. It is not known whether different inspiratory triggering sensitivity settings of ordinary ventilators have different effects on actual ventilation, gas exchange and hemodynamics during resuscitation.

**Methods:**

18 pigs enrolled in this study were anaesthetized and intubated. Continuous chest compressions and mechanical ventilation (volume-controlled mode, 100% O_2_, respiratory rate 10/min, and tidal volumes 10ml/kg) were performed after 3 minutes of ventricular fibrillation. Group trig-4, trig-10 and trig-20 (six pigs each) were characterized by triggering sensitivities of 4, 10 and 20 (cmH_2_O for pressure-triggering and L/min for flow-triggering), respectively. Additionally, each pig in each group was mechanically ventilated using three types of inspiratory triggering (pressure-triggering, flow-triggering and turned-off triggering) of 5 minutes duration each, and each animal matched with one of six random assortments of the three different triggering settings. Blood gas samples, respiratory and hemodynamic parameters for each period were all collected and analyzed.

**Results:**

In each group, significantly lower actual respiratory rate, minute ventilation volume, mean airway pressure, arterial pH, PaO_2_, and higher end-tidal carbon dioxide, aortic blood pressure, coronary perfusion pressure, PaCO_2_ and venous oxygen saturation were observed in the ventilation periods with a turned-off triggering setting compared to those with pressure- or flow- triggering (all *P*<0.05), except when compared with pressure-triggering of 20 cmH_2_O (respiratory rate 10.5[10/11.3]/min vs 12.5[10.8/13.3]/min, *P* = 0.07; coronary perfusion pressure 30.3[24.5/31.6] mmHg vs 27.4[23.7/29] mmHg, *P* = 0.173; venous oxygen saturation 46.5[32/56.8]% vs 41.5[33.5/48.5]%, *P* = 0.575).

**Conclusions:**

Ventilation with pressure- or flow-triggering tends to induce hyperventilation and deteriorating gas exchange and hemodynamics during CPR. A turned-off patient triggering or a pressure-triggering of 20 cmH2O is preferred for ventilation when an ordinary inpatient hospital ventilator is used during resuscitation.

## Introduction

The goal of ventilation during cardiopulmonary resuscitation (CPR) is to provide sufficient oxygenation and removal of carbon dioxide (CO_2_) to improve tissue oxygenation and acidosis. Apart from high-quality chest compressions, hyperventilation has gained increasing attention in recent years because it may negatively affect outcomes in cardiac arrest [1–3]. Manual ventilation with respiratory rates greater than 20 breaths/ min is common during CPR from cardiac arrest [4–6]. An automated transport ventilator (ATV) was found to be as effective as a bag valve mask to deliver ventilation during CPR once the airway is secured [7, 8], and it is recommend by the current guidelines for prolonged resuscitation [9]. In addition, using of ATV may help to avoid uncontrolled ventilation and improve the quality of chest compressions [10, 11]. However, mechanical ventilation by an ordinary automated ventilator rather than an ATV is quite common during resuscitation in actually clinical practice especially in inpatient hospital settings.

Different from the totally time-triggered mandatory breaths of Intermittent Positive Pressure Ventilation (IPPV) in ATV, the IPPV mode in most hospital-based ventilators (such as volume controlled mode) works with time- and patient-triggering simultaneously in order to increase the patient-ventilator synchrony [12]. Patient triggering systems such as pressure- and flow-triggering cannot be turned off in most of the commonly used modern ventilators. Except for respiratory rate (RR), using other ventilatory parameters (e.g. minute ventilation, peak pressure limit) during advanced life support is not recommend under current guidelines [13]. We observed in clinical practice that the changes in airway pressure and airflow induced by chest compressions during CPR may lead to abnormal inspiratory triggering and cause remarkably high ventilation rates. The effects of different inspiratory triggering sensitivity settings of ventilators on actual ventilation during CPR was seldom reported. Additionally, we wanted to see if no triggering was of any benefit in comparison to pressure- or flow- triggering. In this study we investigated the influence of different inspiratory triggering settings using typical hospital-based ventilators on actual ventilation, gas exchange and hemodynamics in a porcine model of cardiac arrest.

## Materials and methods

### Animal preparation

With approval of the ethics committee for animal experiments at Peking Union Medical College Hospital, the study was performed on a total of 18 domestic pigs (weight, 31.1±1.8 kg).

The animals were fasted overnight and premedicated with one dose of intramuscular pentobarbital sodium (30 mg/kg), diazepam (0.3 mg/kg) and atropine (0.02 mg/kg). The animals were then placed supine in a U-shaped fixing frame. An intravenous catheter was inserted into a lateral ear vein and followed by propofol infusion (2–3 mg/kg/hr) to maintain anesthesia. Each pig was intubated with a size 6.5 endotracheal tube and then mechanically ventilated with room air, using volume-control mode (Hamilton-G5, Hamilton Medical AG; Bonaduz, Switzerland), with a tidal volume of 10 mL/kg and a RR adjusted to maintain partial pressure of end-tidal carbon dioxide (P_ET_CO_2_) at 35~40 mmHg and pulse oxygen saturation > 95%. Arterial blood gases (Gem 3000; Instrumentation Laboratory, Bedford, MA) were analyzed to verify the baseline conditions. A warming blanket was used to maintain the animal’s temperature between 36–38°C.

The right internal jugular vein and left common carotid artery were surgically exposed and cannulated for right atrial pressure (RAP) and central aortic blood pressure (AoP) measurements, as well as blood sampling. The left external jugular vein was also fitted with a catheter which provided access for ventricular fibrillation induction. Normal saline solution at 5ml/kg/hr was infused prior to the induction of ventricular fibrillation to maintain RAP between 3 and 5 mm Hg. Electrocardiograms, AoP and RAP were continuously monitored and recorded by a T8 Mindray monitor (Mindray Biological Medical Electronic Co, Ltd, Shenzhen, China). Coronary perfusion pressure (CPP) was calculated as the gradient between AoP and RAP during the decompression phase of chest compressions. RR, minute ventilation volume and airway pressure were continuously recorded by a BIOPAC MP-150 system via a piezometric tube and a flow sensor linked to the endotracheal tube.

### Experimental protocol

**Fig 1:**
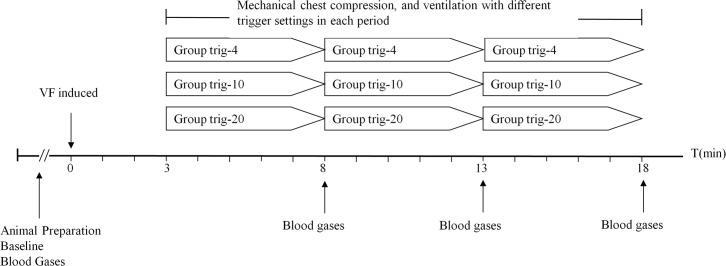
Experimental protocol. VF, ventricular fibrillation.

Ventricular fibrillation was induced by 24V/50Hz AC current with a right ventricular internal-pacing electrode. After 3 minutes of untreated ventricular fibrillation without any ventilation, continuous chest compressions via a mechanical CPR device (WISH-SL-FS-A, Wuhan, China) were started at a rate of 100 compressions/min and a depth of 30% of the anteroposterior chest diameter. Mechanical ventilation (Hamilton-G5) was performed simultaneously for three periods of 5 minutes each, using a volume-controlled mode with a constant flow of 30L/min, zero end-expiratory pressure, FiO_2_ 1.0, RR 10/min, tidal volume of 10 mL/kg, I:E 1:2, and the upper airway pressure limit was set to 60 cmH_2_O (Fig 1). According to the different inspiratory triggering sensitivity settings, the animals were divided into three groups. In the first group of six pigs (Group trig-4), three periods (5 minutes each) of mechanical ventilation were characterized by a pressure-triggering sensitivity of 4 cmH_2_O, a flow-triggering sensitivity of 4 L/min, and turned-off triggering respectively. Similar to Group trig-4, six pigs in Group trig-10 were ventilated with a pressure-triggering of 10 cmH_2_O, a flow-triggering of 10 L/min and turned-off triggering, while six pigs in Group trig-20 were ventilated with a pressure-triggering of 20 cmH_2_O, a flow-triggering of 20 L/min and turned-off triggering. Six sequences in each group were generated due to random assortment of the three different triggering settings, and each animal in each group was ventilated in accordance with one of the six sequences. Arterial and central venous blood gas samples were drawn at baseline and at the endpoint of each 5 minute ventilation period. The animals were finally sacrificed by infusion of potassium chloride at the end of the experiment. Respiratory and hemodynamic parameters at last minute of each period were collected, and the mean values were analyzed.

No.1 Turned-off ---- Pressure-triggering ---- Flow-triggering
No.2 Turned-off ---- Flow-triggering ---- Pressure-triggering
No.3 Pressure-triggering ---- Turned-off ---- Flow-triggering
No.4 Pressure-triggering ---- Flow-triggering ---- Turned-off
No.5 Flow-triggering ---- Turned-off ---- Pressure-triggering
No.6 Flow-triggering ---- Pressure-triggering ---- Turned-off

### Statistical analysis

Non-parametric tests were applied in this study due to the small sample size. Statistical analysis was performed with SPSS 17.0 for Windows (SPSS, Inc.), using the Mann-Whitney *U* test or Kruskal-Wallis *H* test. Continuous variables are expressed as median (25th/75th percentiles) except the animals’ weight. Statistical significance was fixed at a *P* value of less than 0.05.

## Results

Data from all 18 animals were included in the analysis. The results of respiratory parameters, hemodynamic parameters, and blood gases analyses were similar in the three groups at baseline (Table 1).

**Table 1:** Physiological parameters at baseline.

Variables	Group trig-4	Group trig-10	Group trig-20	*P* value
RR (breaths/min)	17.5(15.8/18.8)	17.5(15.8/18.5)	17.5(15.8/20.3)	0.954
MV (L/min)	5.7(5.1/6.1)	5.7(5.1/6.1)	5.2(5.0/6.4)	0.884
AwP (cm H_2_O)	5.0(4.4/5.8)	5.2(4.5/6.3)	5.3(4.2/5.9)	0.911
P_ET_CO_2_ (mm Hg)	36(35.8/38.5)	37(37/39.3)	38.5(37.5/39.3)	0.218
AoP (mm Hg)	108.7(104.8/112.2)	115.2(98.5/124)	109.5(100.1/117.7)	0.696
RAP (mm Hg)	4(3/4.3)	3.5(2/5)	3.5(2.8/5)	0.905
CPP (mm Hg)	94(90.8/103.8)	102(88/111.8)	98(87/102.8)	0.726
PaO_2_ (mm Hg)	100(92.8/105.3)	99(90.3/109.5)	96(92/108.3)	0.960
PaCO_2_ (mm Hg)	41.5(39.3/42.3)	40.5(38.5/43.3)	41(37.5/44.3)	0.964
pH arterial	7.44(7.40/7.47)	7.44(7.39/7.47)	7.44(7.39/7.48)	0.990
PvO_2_ (mm Hg)	43.5(38/46)	40.5(39/47.3)	40(34.8/47.5)	0.801
PvCO_2_ (mm Hg)	44(42.3/48)	44(40.5/48.5)	42.5(40.8/46.5)	0.926
pH venous	7.46(7.40/7.47)	7.41(7.37/7.46)	7.42(7.39/7.47)	0.607
SvO_2_ (%)	74(67.8/77.3)	73(67.8/78.8)	70.5(65/74.8)	0.585

RR, Respiratory rate; MV, Minute ventilation volume; AwP, Mean airway pressure; P_ET_CO2, Partial pressure of end-tidal carbon dioxide; AoP, Aortic blood pressure; RAP, Right atrial pressure; CPP, Coronary perfusion pressure; SvO_2,_ Venous oxygen saturation.

**Fig 2:**
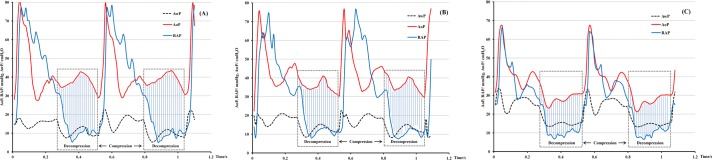
Aortic blood pressure, right atrial pressure and mean airway pressure during the inspiratory phase of an animal in Group trig-20. The period indicated by dotted box is the decompression phase of chest compressions. Coronary perfusion (shadow area) is indicated by the area between red and blue line in the dotted box. (A), Turned-off triggering. (B), A pressure-triggering of 20 cmH_2_O. (C), A flow-triggering of 20 L/min.

In each group, turned-off triggering provided a RR identical to the set value, while the pressure- and flow-triggering could induce significantly higher actual RR with the exception of a pressure-trigger of 20 cmH_2_O (all *P***<**0.05, Table 2). Except for the pressure-trigger of 20 cmH_2_O, significantly higher minute ventilation volumes, mean airway pressures, and lower P_ET_CO_2_, AoP and CPP were also induced by the pressure- and flow-triggering settings compared to turned-off triggering (all *P***<**0.05, Fig 2).

**Table 2:** Results of Respiratory Monitoring and Hemodynamics.

	RR (breaths/min)	MV (L/min)	AwP (cm H_2_O)	P_ET_CO_2_ (mmHg)	AoP (mmHg)	RAP (mmHg)	CPP (mmHg)
**Group trig-4**							
OFF	10.5(10/11.3)	4.8(4.3/5.5)	7.2(6.0/7.8)	28.5(26.3/32.5)	46.8(43.3/52)	30(25.5/30)	29.2(26.4/32.3)
Pressure	26.5(24.5/27.5)	8.6(7.8/9.6)	13.5(11.5/14.9)	20.5(17.3/22.5)	40(34.7/43.9)	25.5(24.3/31.5)	21(18.1/27)
*P* value^a^	0.004	0.004	0.004	0.005	0.024	0.198	0.037
Flow	26.5(22.8/28.0)	8.4(7.9/9.5)	13.0(10.8/14.2)	19.5(17/22.8)	39.2(36.4/45.5)	23.5(22/27.5)	21.6(19.2/24.9)
*P* value^b^	0.004	0.004	0.004	0.006	0.037	0.053	0.037
**Group trig-10**							
OFF	11(10/11.3)	4.9(4.6/5.2)	6.6(6.0/8.1)	28.5(25.5/32.8)	47(43.7/50.2)	28.5(26.5/30.5)	30.1(23.3/32.8)
Pressure	20.5(19/22.5)	6.8(5.6/8.5)	12.4(10.8/14.2)	23(19/24.3)	39.4(38.1/43.7)	24.5(21.5/28.3)	21(19.4/25)
*P* value^a^	0.004	0.004	0.004	0.008	0.010	0.077	0.025
Flow	23(21.8/25)	7.5(6.6/8.8)	13.0(12.1/14.2)	22(20/24.5)	40.7(38/43.9)	23(20.8/26.3)	21.7(17.8/23.1)
*P* value^b^	0.004	0.004	0.004	0.010	0.025	0.029	0.010
**Group trig-20**							
OFF	10.5(10/11.3)	4.8(4.4/5.2)	6.9(6.7/7.6)	28.5(26.8/30.5)	45.2(39.6/49.2)	27.5(23.8/33.5)	30.3(24.5/31.6)
Pressure	12.5(10.8/13.3)	5.2(4.7/5.7)	8.3(7.2/9.9)	26.5(24.8/27.3)	41(38.9/42.9)	25(22/30.3)	27.4(23.7/29)
*P* value^a^	0.070	0.127	0.065	0.076	0.149	0.419	0.173
Flow	22.5(21.8/23)	7.1(6.5/8.1)	12.3(11.5/13.2)	21.5(18.8/24.3)	37(35.2/39.2)	24(22.3/26)	20.9(18/23.8)
*P* value^b^	0.003	0.004	0.004	0.013	0.025	0.091	0.016

RR, Respiratory rate; MV, minute ventilation volume; AwP, Mean airway pressure; P_ET_CO2, partial pressure of end-tidal carbon dioxide; AoP, aortic blood pressure; RAP, right atrial pressure; CPP, Coronary perfusion pressure.

^a^ Turned-off triggering versus Pressure-triggering

^b^Turned-off triggering versus Flow-triggering.

In each group, significantly lower arterial pH, PaO_2_, and higher PaCO_2_ were observed in the periods with turned-off triggering compared to those with pressure- or flow- triggering (all *P***<**0.05), except in the comparison with pressure triggering in Group trig-20 (Table 3). The venous blood gas analyses showed that most of the differences of venous pH, PvO_2_ and PvCO_2_ between the different triggering settings were not statistically significant. Turned-off triggering was related to higher venous oxygen saturation (SvO_2_) than those of pressure- or flow- triggering settings in each group (all *P***<**0.05), with the exception of a pressure-triggering of 20 cmH_2_O (46.5[32/56.8] % vs 41.5[33.5/48.5] %, *P* = 0.575).

**Table 3:** Results of Arterial and Venous blood Gas Analysis.

	PaO_2_ (mmHg)	PaCO_2_ (mmHg)	pH arterial	PvO_2_ (mmHg)	PvCO_2_ (mmHg)	pH venous	SvO_2_ (%)
**Group trig-4**							
OFF	87(77.5/99.8)	48(43.3/57.5)	7.30(7.21/7.35)	30(25/33.8)	63(53.8/68.5)	7.21(7.17/7.26)	46(32.5/58.3)
Pressure	103.5(98.5/121.8)	23.5(18/28)	7.50(7.44/7.53)	24.5(19.3/27.8)	58(53/68.5)	7.23(7.16/7.26)	22.5(19.5/34.8)
*P* value^a^	0.016	0.004	0.004	0.078	0.629	0.747	0.030
Flow	106(98.5/115)	21(19.3/32.5)	7.48(7.43/7.54)	23(19.8/28.5)	57(53.8/66)	7.22(7.14/7.26)	24.5(20.8/33.8)
*P* value^b^	0.020	0.004	0.004	0.077	0.687	0.809	0.045
**Group trig-10**							
OFF	85(70/98.8)	49(43.8/56)	7.28(7.22/7.31)	29(25.8/32)	60(53.3/73)	7.25(7.17/7.30)	48.5(34/57.3)
Pressure	101.5(95/118.3)	26(22.8/30.3)	7.46(7.41/7.55)	22.5(19.3/26)	67(55.5/68.5)	7.20(7.16/7.28)	24.5(20/34)
*P* value^a^	0.045	0.004	0.004	0.037	0.872	0.522	0.016
Flow	110.5(102.5/122.8)	24.5(19.8/35.3)	7.50(7.46/7.52)	23(19.5/28.5)	59.5(56.5/65.8)	7.24(7.19/7.30)	27(19.3/34.8)
*P* value^b^	0.008	0.004	0.004	0.054	1.0	0.936	0.025
**Group trig-20**							
OFF	84(78/98.3)	50.5(48.5/57.8)	7.28(7.22/7.31)	28.5(25.3/34.5)	60(53.3/63)	7.21(7.14/7.25)	46.5(32/56.8)
Pressure	94.5(82.5/99.8)	46.5(43.8/48.8)	7.32(7.23/7.37)	26(21/30.3)	61(52.3/68)	7.19(7.15/7.30)	41.5(33.5/48.5)
*P* value^a^	0.378	0.030	0.394	0.378	0.810	0.810	0.575
Flow	114(98.3/132)	28(20.3/31.8)	7.49(7.39/7.52)	22.5(19.5/25.3)	57.5(52/65.5)	7.21(7.17/7.30)	23.5(19.8/38)
*P* value^b^	0.016	0.004	0.004	0.077	0.873	0.574	0.037

SvO_2,_ venous oxygen saturation.

^a^ Turned-off triggering versus Pressure-triggering

^b^Turned-off triggering versus Flow-triggering.

**Fig 3:**
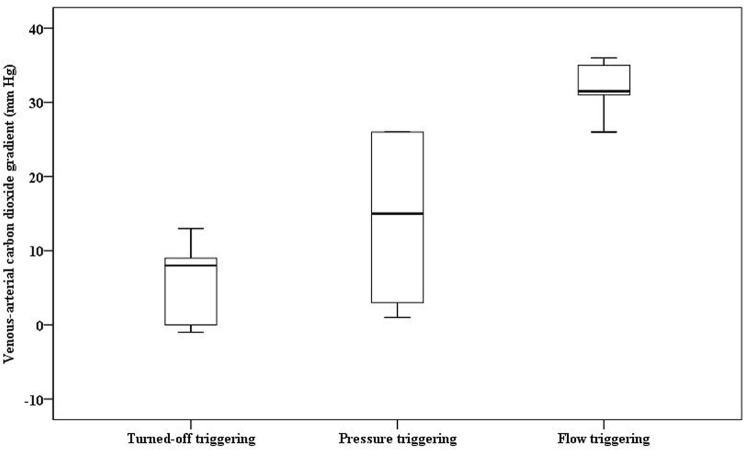
Venous-arterial CO_2_ gradients in Group trig-20 (median, 25/75% percentiles, min/max) [mmHg].

In Group trig-20, the venous-arterial CO_2_ gradients for turned-off triggering, pressure-triggering and flow-triggering were 8(-0.25/10) mmHg, 15(2.5/26) mmHg and 31.5(29.8/35.3) mmHg respectively (Fig 3). The venous-arterial CO_2_ gradients for flow-triggering were significantly higher than those of turned-off triggering (*P* = 0.006). The comparison between turned-off triggering and pressure-triggering did not show statistical significance (*P* = 0.148).

## Discussion

This study investigated ventilation via a typical hospital ventilator using different inspiratory triggering settings during CPR. Compared to the turned-off patient triggering, most settings of pressure- or flow-triggering induced hyperventilation through a high RR, with the exception of pressure-triggering at 20 cmH_2_O. Though a higher PaO_2_ was observed, elevated mean airway pressures and deteriorating CPP, P_ET_CO_2_ and SvO_2_ seen with these settings indicated adverse prognoses. A pressure-triggering of 20 cmH_2_O had similar ventilation volume, gas exchange and hemodynamics as compared to turned-off triggering, and could be an alternative choice if an ATV or turned-off patient triggering was not available in clinical practice.

Excessive ventilation is strongly suggested to be avoided in the current guidelines, due to its adverse effects increasing intrathoracic pressure, decreasing venous return, coronary perfusion, and diminishing cardiac output and survival [1, 13]. Avoidance of hyperventilation during CPR is a broad consensus, though clinical studies confirming the association between hyperventilation and cardiac arrest outcomes are limited. However, hyperventilation commonly occurs during CPR with RR exceeding 10 breaths/min 63% of the time and exceeding 20 breaths/min 20% of the time, despite guideline recommendations [6]. The precipitating factors of hyperventilation are usually multifactorial including an adrenaline-driven arousal response, resuscitator inexperience, and CPR delivered at off-hours [6, 14, 15]. RR by manual ventilation could even reach 37 breaths/min with advanced airways, and providers often fail to ventilate at recommended rates even after retraining [1, 4, 16].

The use of a mechanical ventilator during CPR can theoretically eliminate hyperventilation by totally controlling ventilation rates and tidal volumes without patient triggering. An ATV is recommended in the guidelines as one of the ventilation alternatives for prolonged resuscitation efforts to provide adequate ventilation and allow resuscitators to perform other tasks [9], while the automatic mode of the oxygen-powered, flow-limited ventilator should not be used due to its adverse effects on venous return and forward blood flow [17]. The mechanical properties of typical hospital-based ventilators are usually superior to transport ventilators, and these ordinary ventilators are often involved in CPR due to their availability and convenience in our clinical practice. However, patient-triggering cannot be turned off in most modern ventilators (e.g. Puritan Bennette 7200ae & 840, MAQUET servo-i, Drager infinity C500, and GE Engstrom carestation, among others). The current guidelines do not give any specific ventilatory recommendations except for the RR with a secured airway, and, to the best of our knowledge, there are no studies focusing on different inspiratory triggering settings during CPR until now.

In general, the maximum adjustable range of pressure- or flow-triggering of most modern ventilators is 20 cmH_2_O or L/min. This study demonstrated that a pressure-triggering of 20 cmH_2_O rather than a flow-triggering of 20 L/min could effectively control hyperventilation. Because of design characteristics, flow-triggering provides more sensitive triggering and relatively shorter time delay than pressure-triggering [18, 19]. It’s reasonable to surmise from our study that airway pressures are usually between -10 and -20 cmH_2_O, while airflow is more than 20 L/min in most cases during the decompression phase of chest compressions. Adverse effects of hyperventilation were verified in this fundamental research again. High ventilation rates greater than 20 breaths/min induced high minute ventilation volume, increased mean airway pressure, and decreased CPP in this study.

However, contradictory viewpoints and research challenging the adverse effects of hyperventilation or high airway pressures during CPR have emerged in recent years [20–22]. Higher mean airway pressure was found to be associated with higher ROSC in clinical settings [22]. A novel ventilation mode (Chest Compression Synchronized Ventilation, CCSV) which increases cyclic intrathoracic pressure differences and avoids negative airway pressure shows better effects on oxygenation and hemodynamics compared to IPPV [23]. Meanwhile, the need for artificial ventilation in the first minutes of CPR has also been challenged in the past several years [24]. Passive ventilation such as continuous positive airway pressure (CPAP) with pure oxygen via a Boussignac device or CPAP plus pressure support ventilation, was found to an effective alternative to mechanical ventilation with similar or superior gas exchange [25, 26]. Nevertheless, positive-pressure ventilation is still the “gold standard” of ventilation during CPR, though further investigations are needed to clarify the specific effects of ventilation volume and airway pressure on the outcomes of CPR.

As regards gas exchange in this study, high RR caused by pressure- and flow-triggering were correlated with decreased P_ET_CO_2_, alkalosis and better arterial oxygenation. Arterial blood gas values were often used as measurable indicators of resuscitation effects in may studies of CPR [27–29]. However, PaCO_2_ and PaO_2_ are simultaneously influenced by ventilation and pulmonary blood flow [30]. With significantly decreasing pulmonary blood flow (increasing ventilation/perfusion ratio), PaO_2_ increases and PaCO_2_ decreases, approaching the composition of inspired gas, which is opposite to conventional practice [30]. Similar to the current study, increased PaO_2_ and decreased PaCO_2_ during low blood flow states of CPR, may indicate not only adequate or excessive ventilation but also the negative effects of deteriorating blood flow. In general, oxygen delivery to the heart and brain is limited by blood flow rather than by arterial oxygen content during CPR [9, 31, 32].

Venous blood gases are thought to be more useful than arterial blood gases to assess perfusion during resuscitation, because they are less affected by ventilation changes [29, 30]. Actually, similar values of venous blood gases analysis were observed in most groups in our study, except that controlled ventilation rates with turned-off triggering or pressure-triggering of 20 cmH_2_O showed better SvO_2_. However, the same phenomenon was also observed in human CPR [33]. Subjects with restoration of spontaneous circulation (ROSC) demonstrated significantly different values of venous PH, PO_2_, and PCO_2_ during resuscitation, while a higher SvO_2_ indicated high rate of ROSC from cardiac arrest.

In addition to decreased CPP and SvO_2_, flow-triggering also correlated with increased venous-arterial CO_2_ gradients. Low blood flow states such as during chest compressions or shock, PaCO_2_ positively correlates with blood flow while venous PCO_2_ negatively correlates with blood flow [29, 34]. The increased venous-arterial CO_2_ gradients in this study indicated decreased blood flow during CPR, and suggest again that hyperventilation due to flow-triggering deteriorates cardiac output and tissue blood flow.

Our study has several limitations. First, the sample of animals was relatively small and outcomes of resuscitation efforts such as ROSC could not be detected and compared because of the cross-over design. Second, 5 minutes for each ventilation period was relatively short. That was chosen because the differences of respiratory parameters, gas exchange and hemodynamics between different triggering settings were significant after a ventilation duration of 5 minutes, and prolonged CPR would induce rapid deterioration of artificial circulation. Finally, an ATV was not involved in our study to serve as a point of comparison with our hospital-based ventilators. When the triggering of a ventilator is turned off, its ventilation process is basically identical to that of an ATV. Furthermore, the purpose of this study was to evaluate the feasibility of using different inspiratory triggering settings in an ordinary ventilator during CPR.

In conclusion, for ordinary automated ventilators found in inpatient hospital settings, pressure-triggering of 4 or 10 cmH_2_O and flow-triggering are improper for ventilation during CPR, due to their induction of hyperventilation leading to the deterioration in gas exchange and hemodynamics. Turned-off patient triggering is preferred, and a pressure-triggering of 20 cmH_2_O could be a viable alternative if turned-off triggering is not available.

## Supporting information

S1 File Relevant data underlying the findings described in manuscript. (XLSX)
